# Lunatic Asylums

**Published:** 1840

**Authors:** 


### LUNATIC ASYLUMS.

For the information of such of our brethren, as reside in places remote from the few Asylums for the insane which have as yet been erected in the Valley of the Mississippi, we should be happy to speak of them in detail. We know so little, however, of that in Tennessee, of which Dr. Kelley is superintendent, that we can but indicate its existence, and ask for information.

With that established by the State of Kentucky, in the city of Lexington, our acquaintance, in former days, was quite intimate; and we have reason to believe, that it is now, what it then was, a well provided and well ordered establishment. We have not, however, received any recent report of its progress or present condition, and cannot, therefore, say more at present, than that it merits the confidence of those who are called upon to provide for insane friends.

Of the OHIO LUNATIC ASYLUM, in Columbus, we know much more, having lately received the Annual Report to the Legislature, by its Directors and Superintendent. This is the first report, made after the opening of the Infirmary, and comprises the year from November, 1838, to November, 1839.

During that time, 157 patients were admitted, concerning whom, we extract the following tabular view, the RECAPITULATION of one much more extended.

Whole number of patients admitted into the Asylum from the 30th November, 1838, to the 15th November, 1839, - - - - - - - - 157

| | | |
|---|---|---|
| Males, - - - - - - - - - - - - - | 87 | |
| Females, - - - - - - - - - - - | 70—157 | |
| Old cases, - - - - - - - - - - - | 114 | |
| Recent do* - - - - - - - - - | 43—157 | |
| Paupers, - - - - - - - - - - - | 125 | |
| Pay patients, - - - - - - - - - | 32—157 | |
| Single, - - - - - - - - - - - - | 88 | |
| Married, - - - - - - - - - - - | 56 | |
| Widows, - - - - - - - - - - - | 11 | |
| Widowers, - - - - - - - - - | 2—157 | |

| | | |
|---|---|---|
| Discharged during the year. | Recovered, - | 27 |
| | Incurable, - | 5 |
| | Idiotic, - - | 2 |
| | Eloped, - - | 1 |
| | Died, - - - | 8— 43 |
| | Males, - | 61 |
| | Females - | 53—114—157 |

Per cent. of recoveries on recent cases in 8 months, - - - 71.43

Of those remaining in the Asylum,

| | |
|---|---|
| The prospect seems to be entirely favorable for | 15 |
| " " favorable " | 15 |
| " " doubtful " | 34 |
| unfavorable " | 50—114 |

*Duration of insanity before admission.*

Less period than one year, - - - 43

* Cases are denominated RECENT OR CURABLE when the duration of the disease is less than ONE YEAR before the admission of the patient. Institutions differ much in regard of this RULE, which is deserving of notice, as the result will be materially changed where the periods of 3, 6 or 9 months are adopted.

| | |
|---|---|
| From one year to five years, - - - | 67 |
| " five to ten years, - - - - | 22 |
| " ten to twenty years - - - - | 16 |
| " twenty to thirty years, - - | 6 |
| Unknown, - - - - - - - - - - | 3—157 |

*Ages of the patients when admitted.*

| | |
|---|---|
| Under twenty years, - - - - - - | 7 |
| Between twenty and thirty, - - - | 71 |
| Between thirty and forty, - - - - | 41 |
| Between forty and fifty, - - - - | 20 |
| Between fifty and sixty, - - - - | 14 |
| Between sixty and seventy, - - - | 4—157 |

*Supposed remote or exciting causes.*

| | |
|---|---|
| Intemperance, - - - - - - - - | 7 |
| Domestic affliction, - - - - - | 6 |
| Puerperal, - - - - - - - - - | 13 |
| Ill health of various kinds, - - - | 14 |
| Loss of friends, - - - - - - - - | 4 |
| Matrimonial perplexities, - - - - | 4 |
| Fright, - - - - - - - - - - - | 3 |
| Intense application, - - - - - - | 3 |
| Jealousy, - - - - - - - - - - - | 2 |
| Disappointed Love, - - - - - - | 10 |
| Epilepsy, - - - - - - - - - - - | 9 |
| Injuries of the head, - - - - - | 5 |
| Constitutional, - - - - - - - - | 10 |
| Disappointment and mortification, - | 10 |
| Masturbation, (*produced or perpetuated by the practice*,) - - - - - - - | 16 |
| Fear of want, loss of property, &c., | 7 |
| Ill treatment from parents or guardians, | 2 |
| Religious excitement and anxiety, including perplexity, exaltation, enthusiasm, fanaticism, doubt and fear of future punishment, - - - - - - | 15 |
| Unknown, - - - - - - - - - - | 17—157 |

*Species of insanity.*

| | |
|---|---|
| Mania, - - - - - - - - - - | 101 |

| | | |
|---|---|---|
| | " melancholic variety, | 17 |
| | " epileptic " | 12 |
| | " homocidal, | 4 |
| | Moral insanity, | 10 |
| | Incoherence or dementia, | 10 |
| | Idiotism or imbecility, | 3—157 |

*Occupations.*

| | | | |
|---|---|---|---|
| Laborers, | 16 | Farmers, | 27 |
| Millwrights, | 2 | Blacksmiths, | 3 |
| Brick-mason, | 1 | Brewer, | 1 |
| Carpenters, | 7 | Shoemakers, | 3 |
| Students, | 3 | Teachers, | 3 |
| Tailors, | 3 | Lawyers, | 2 |
| Merchant, | 1 | Sadler, | 1 |
| Cooper, | 1 | Weaver, | 1 |
| Collier, | 1 | Clerk, | 1 |
| Potter, | 1 | Preacher, | 1 |
| Engineer, | 1 | Musician, | 1 |
| Sea Captain, | 1 | | |

*Places of nativity.*

| | | | |
|---|---|---|---|
| Ohio, | 44 | Pennsylvania, | 24 |
| New Jersey, | 4 | New York, | 8 |
| New Hampshire, | 5 | North Carolina, | 1 |
| Connecticut, | 6 | Vermont, | 9 |
| Virginia, | 12 | Kentucky, | 3 |
| Delaware, | 1 | Maryland, | 2 |
| Massachusetts, | 8 | Tennessee, | 1 |
| Germany, | 9 | Ireland, | 7 |
| England, | 8 | Scotland, | 3 |
| Rhode Island, | 1 | | |

*Admitted and discharged.*

| Months. | Admitted. | Discharged. |
|---|---|---|
| November, | 2 | – |
| December, | 25 | – |
| January, | 14 | – |
| February, | 4 | 4 |
| March, | 3 | 3 |
| April, | 5 | 1 |
| May, | 13 | – |
| June, | 16 | – |
| July, | 30 | 2 |
| August, | 13 | 4 |
| September, | 13 | 11 |
| October, | 10 | 13 |
| November, | 9 | 5 |
| | 157 | 43 |

The edifice is situated one mile east of Columbus in the midst of ample grounds, and in a healthy locality. It is the most extensive building, we suppose, in the Valley of the Mississippi, and fashioned after the best models of the Eastern States. The whole discipline of the establishment is mild and paternal. All restraint, not *absolutely* necessary, is avoided, and every moral influence is pressed into the treatment of the unfortunate inmates. The superintendent, Dr. Awl, has made the management of Lunatic Asylums a special study, and is unwearied in his efforts, for the comfort and care of all the patients—facts which we do not derive from the Report, so much as from other sources, and especially personal observation. It is proper to state, for the benefit of persons at a distance, that Columbus may be reached, either from the north or south, by the Ohio and Erie canal, which, as it crosses the State from Cleveland to Portsmouth, is connected with Columbus. D.

